# Supplementary material for: Has food security in the EU countries worsened during the COVID-19 pandemic? Analysis of physical and economic access to food
Source: PLoS One. 2024 Apr 17;19(4):e0302072. doi: 10.1371/journal.pone.0302072 (PMC11023501; doi:10.1371/journal.pone.0302072)
Supplement: Attachment — Submitted filename: Response to Reviewers.docx [file pone.0302072.s001.docx]

**Answers to Review Comments to the Author**

Dear Reviewers, we would like to thank you very much for your time and valuable comments and suggestions to improve our paper. We included all of them in our manuscript. We hope that now, it is much more clear and consistent. Many parts of the paper were rewritten. Some parts were also removed, according to Reviewers suggestions, or modified in order to improve their quality.

Our answers to specific comments are included below.

**Reviewer #1:**

Why is the Covid-19 pandemic viewed as a climate-environmental shock by the author as one must conclude from the text in ll 59-64? It has been a public health phenomenon, not related to climate. It affected the environment, but not in the context of the climate change as the text would suggest.

The phrase in lines 59-64 has been modified to avoid such a misleading expression. See line: 62-63

l. 94; do you mean “food access”? Not sure what is meant by “economic food access” (what is the difference between food access and economic food access?). Is it “affordability”? Clarify. See your discussion in ll 180-188.

This has been clarified. See line: 94-100

l. 104; do you have the data to make inferences about the “healthy diet”? Or, is the focus only on household ability to purchase food (affordability) and disruption of trade flows?

Thank you for your comment. Indeed, our research focuses on a diet with meat or its equivalent (not exactly “a healthy diet”), by using an indicator published by Eurostat: *capacity to afford a meal with meat, chicken, fish or vegetarian equivalent every second day*. Whereby the focus is on “capacity to afford” rather than the type of food (meat or vegetables).

This is a common measure of household food insecurity in the EU which refers to access to enough food for an active, healthy life (e.g. Loopstra). It does not reflect all aspects of proper nutrition, but it is available for all EU countries, and there are no differences related to data collection methodology.

In the article, we have changed the “healthy diet” to the “diet with meat, chicken, fish or equivalent” according to the Eurostat data, and added relevant explanations ion the entire file.

Section 2; you discuss the actual epidemics (for example, the bird flu), not the threat of epidemics. Use the term “epidemic”.

This has been done corrected.

Ll 141 and following. There was no outbreak of BSE in the USA. There were very few cases and they were handled expeditiously. What did happen, was the ban of US beef in several major importing countries and, given the dependence on beef exports, the cattle and beef industry in the US was affected.

This comment has been implemented. See line:141-144

Ll 150-151; advice caution in making such statements. Meat affordability is a problem in low income countries, which are more likely to consume fowl, rather than pork. The observation may be applicable to the middle-income countries. In low income countries, consumers depend on plant protein sources.

This has been clarified. See line:152

l. 159; was it the loss of income or the loss of purchasing power due to inflation? What has caused incomes to decline? Loss of jobs? Clarify.

This has been clarified See line:160-161

l. 159 and following; use consistently the same terminology; is Covid-19 the same as SARS…? If yes, choose one term and use in the paper. Similarly, l. 170, Covid-19 pandemic or crisis? Use one term only.

This has been done in entire paper.

Ll 211-213; do not use one-sentence paragraphs. Merge with the following paragraph.

The section has been rebuilt. There are no one-sentence paragraphs in the current version of the manuscript.

Ll 213-217; note that now you reversed the order listed in ll 94-101, where the household ability to afford food was the first objective, and the trade flow disruption was the second goal. By consistent in your presentation and analysis. The following text reversed the order again. This is confusing.

The order of research questions given in lines 94-101 has been changed. Now all sections of the manuscript present the same logic order.

Ll 218-222; you seem to mix the analysis of affordability and the trade flows: “…as well as other indicators related to the physical food access (such as trade dynamics)” Please, clarify.

This has been clarified See line:211-218

L. 223 and l. 226; replace the word “variables” by stating clearly what factors you consider. Also, is the word “or” following 2) correct, or should it be “and”.

We replaced the formulation “variables” with “data” and explained in brackets which data we specifically consider.

Ll. 229 and following; the discussion gets confusing. It seems that you are now pondering what to do. This is the section about methodology and by now, it must be clear in your approach what you do and present the data and the methods.

We reformulated the entire paragraph, leaving just an explanation of the approach chosen. See line 261-271.

Comment: The sentence (starts in l. 236): For example, positive error values indicate that COVID-19 contributed to the growth of a given phenomenon during that time, is not clear.

We clarified this sentence and added the equation to explain the forecast error idea. “In the second approach, we assume that Covid-19 could have led to significant changes in the level of variables reflecting the level of food security in EU countries. The direction and strength of the impact of the pandemic can be concluded by comparing two paths of development of the phenomenon: "COVID-19" and "no-COVID-19". Comparison of the observed time paths of the actual levels of variables (COVID-19) with those that would have been expected in the no-COVID-19 scenario defined by the forecasting model allows estimation of the direct impacts of the pandemic. Differences between scenarios are expressed in the form of ex-post forecast errors in a country i and in time t:

Positive error values indicate that COVID-19 contributed to the growth of a given phenomenon, whereas negative values indicate that COVID-19 negatively impacted it.

Comment: l. 239; “most” variables are annual observations or all of them? Such a statement without presenting the data first confuses the reader; it is not clear what you refer to.
After reorganising the section and adding a new table with data description, this should be clearer. See line: 248+

Comment: In l. 239, there is a reference to prices, but the factors named earlier included income, wages, etc. beside prices. The presentation must be reorganised to assure clarity.
In l. 239, we cannot find a reference to prices. Anyway, we reorganised this section to be more concise. See lines 248-271

Comment: l. 280; here the references is made to annual and monthly observations. Clearly separate the section into the presentation of the data and then discuss the applied methodology in the context of the available data because it seems the nature of the data dictates the choice of analytical tools, not the other way round.

Thank you for your comment. We reorganized our section according to your suggestion.

Comment: l. 239 and ll 302-303 present contradiction – are the data since 2000 or since 2005? You need to clearly state what is the length of an observation (monthly or annual for what data) and what data are for what period (2000, after 2005?). A clear presentation of each variable series, with the length of observation, period, and definition of units in a table and supported by a description will greatly improve the coherence of the presentation.
Thank you for your comment. Indeed the data explanation was a bit chaotic. According to Reviver’s advice, we added a new table explaining the data used. We also slightly modified the description. See line:248+

Comment: Table 1 and its description; it appears that the model overestimated (the predicted value > the actual value, so the reported figure is negative) the decrease in non-food trade monthly values in 2020 and underestimated the decrease in 2021. The pattern in forecasting the food trade changes was the same as in the non-food trade in 2020, but less consistent in 2021. This is what Table 1 shows and suggests greater volatility in food trade. One would prefer a model that is consistent (either over- or under-estimates) over a model that yields variable predictions.

The methodology used to assess the impact of COVID-19 on trade is based on a comparison of forecasts made on the basis of data until February 2020 to actual values during the pandemic. The negative values shown in Table 1 (now it is table 2) indicate a negative impact of COVID-19 on trade, while the positive ones indicate a positive impact. The predominance of negative forecast errors (including the sum of errors) indicates that the COVID-19 pandemic led to a decrease in trade and it was impossible to make up for the losses caused by COVID-19 by the end of 2021. The predominance of negative values does not mean that the models are wrong (underestimated or overestimated) but that the shock caused by the pandemic was so strong that it led to structural changes in foreign trade in the forecasting period (pandemic period). The text attempts to clarify these issues.

All figures lack the indication of the Y axis. Please, mark Y-axis on each figure.

Indication of the Y axis on all Figures has been added.

l. 364 and following; there is lack of clarity: the title of Table 1 suggests that the figures in the table are the differences between the actual and predicted values. In l. 362, you suggest that the smaller negative values in the case of food trade indicate that the trade was less affected, but is this the case? We do not know the absolute values (is it in money rems or weight?) of the actual trade to make such an inference. The figures in Table 1 show the difference between the actual and predicted values and ignore the volume. If the actual trade declined from month to month, then even if the differences are small, the actual declines could have been large enough to compromise food security. If the values used in the analysis are in money terms, then the inflation may have hid the size of the decline in weight. There is some relevant discussion missing.

Thank you for this remark. The discussion of data in Figure 1 has been clarified to avoid doubts whether the volume or value of import was analysed.

Ll 362-378; you may want to clearly delineate between your findings and the references to other studies, especially in the context of diet quality. Your analysis does not provide (at least until this point) any measure of trade composition that would indicate the change in quality of the diet.

Thank you for this comment. The paragraph has been reformulated. Diet-conection has been removed. See line: 350-366

Comment: Review your use of the term “trend” or “trends” in various places in the paper. Trend is a statistical term with a specific meaning that, in my view, does not fit the context in which you used it.

In our opinion, in most of the sentences in the manuscript, this term is correctly used. We can distinguish the following patterns in a time series: trend, cycle, seasonal and irregular (random) variation. Frequently, trend and cycle are not split and are treated as trend or trend-cycle. This term is used correctly in the context of the forecasting methodology used (Holts’ and Holt-Winter's models). In a few other places, we changed this term to more neutral (patterns, tendencies, regularities).

Please use Russia-Ukraine war, not “conflict in Ukraine”. The latter is misleading and suggests this is an internal Ukraine problem which is not true.

It has been changed in entire manuscript.

l. 406; re-write.

It was rewritten. See line: 393-395

Ll 407-410; the last sentence, referring to [10], is used as the explanation of your results. However, the connection is tentative because those are two different studies. Results in [10] may coincide with your results, but they do not outright explain them.

We reformulated this sentence, indicating that the price increase (not directly our results) was explained in the literature. See line: 399

L 414 and following; remind the reader what data you use to discuss the basket of consumer goods (from what period?) and the specific method used. Again, caution the reader because the comparison is based on information collected at distant points in time and could have been shaped by several factors. You attribute the observed differences to the Covid-19 pandemic without any hard evidence.

The time frame of the data on analysis the share of food in the basket of goods purchased by the consumer has clarified,while the analysis has been discussed more extensively. Moreover, the time frame of all analyses throughout the study has been presented in more detail in Table 1 that has been added in the Data and methodology section. See lines: 404-421.

Ll436-437; income is not linked to economic growth. Income changes may be linked to economic growth or decline. Be precise.

It has been changed to " general economic situation".

L 437 and following; PPS is affected by each country’s rate of inflation. l. 441; is it -5.1% and -1.1%? The discussion in ll 443-449 is disconnected from the earlier discussion of the PPP. PPP and disposable income are two different concepts and cannot be compared. From the household perspective, it is PPP that decides the household budget constraint and ability to purchase food. Only in this context can you place comments about the disposable income, which although increased, apparently not enough to offset the decline in PPP. The disposable income may have increased due to government policies such as suspending some taxes or direct transfers, but PPP decreased because of the rising price level. Clarify those issues and their distinction.
L 456; you just showed that PPP has declined, despite larger disposable income. It is not clear what is the purpose of the discussion in ll 456-468. You bring a completely new issue, the social depravation without defining what do you mean. I suggest to delete the whole paragraph.

The section is confusing. In L. 482 you mentioned disposable income, but it is PPP that really measures the ability to buy. You concluded the PPP has declined, so how relevant is the increase in the disposable income? You cited a reference earlier with regard to the fiscal policies – I commented on the issue above – a suspension of selected taxes or direct transfers to individuals could have increased the disposable income and partially offset the price increases (captured in PPP deterioration). Consumption of animal protein is your selected indicator, but it is only one factor. Consumers have been documented to substitute one food type for another. For example, increasing the consumption of yogurt or curd provides animal protein and replaces meat. You notice that the period 2010-2013 was different and that period was associated with the global financial crisis of 2009/2010. You can make very tentative statements and I wonder if they really make a meaningful difference in your paper.

The following response applies to comments beginning with L 437- L482.

Thank you for pointing out some inconsistencies regarding the issues of income, salaries and PPP. We have decided to rewrite the mentioned paragraphs to emphasise: 1. the decline in PPP and its income components, including wages; 2. the increase in disposable income as a result of social transfers; 3. the stronger growth in income than the increase in food prices.

Referring to the PPP itself. In principle, it points to the purchasing power of consumers. However, it is of a more general nature, referring to the state of the entire economy. Its decline can, for example, be associated with a decline in state income, hence the differences from the disposable income of the population. We believe that in the short term, the situation of consumers is better reflected by disposable income. And the improvement or deterioration of food security can be evidenced by relating their increase/decrease to changes in food prices.

Regarding the consumption of animal protein - throughout the article we have emphasized more that the indicator we used refers to the possibility of consuming meat, fish or vegetable equivalent. Thus, it refers to protein in general, regardless of its source. In the first version of the article, this was indeed not specified.

The paragraph related to social deprivation was deleted according to suggestion of Reviewer. To sum up: the entire description of this section was rewritten. See line 427-461. We deleted also some subjective indicators like “meeting ends meets”, and added a new subtitle.

**Reviewer #2:**

The authors made a bold attempt to investigate how COVID-19 affected Food Security in the EU. The research is novel in that only few studies have delved into this regional analysis with most of the literature cited carrying out their research at country-level. It is commendable that the Russian-Ukranian war is factored into the approach that the authors engaged.

However, I have strong reservation with the author's attempt to subjectively analyse food security based on consumers' perception. Food security concept has clearly established quantitative and qualitative indicators that can be used and as such should not be based on perception or questions around "make ends meet". In my opinion, this poses a major flaw for the manuscript, hence needing revision. If the authors are unable to primarily examine the food security, the manuscript may be better off without the consumers' perception of financial situation

Thank you for your valuable comment. We agree with it. It was also rised by the other reviewer. We decided to delete the section related to consumer perceptions of financial situation and ability to “make end meets”. We also rewrote entire subchapter. See line 465+

I also suggest that the authors need to rein in their review of literature to make this more centered on the main points of their research and for a better flow.

Thank you for your valuable comment, it might arise from the fact that an analysis of impacts of epidemic shocks on food security has a multidimensional character. We however think that logic of the literature review, in our opinion, is adjusted to this paper scope, and includes:

1) First, effects of past epidemic shocks (BSE,ASF, Avian flue),
2) Then, economic effects of COVID pandemic in general related to income per capita, food shortages, household concerns about capacity to ensure food availability), which further affects analysis of:
3) effects of COVID pandemic on food security in low-income countries and later in developed countries, where we indicate the knowledge gap.

The manuscript may also benefit from grammatical improvements in some areas. For instance, line 105-106. To address the grammatical errors, I suggest authors use a grammar editor tool such as grammarly.

The native speaker proofread the text we also used Gramarrly business to check for minor language mistakes.

If the authors are unable to primarily examine the food security, the manuscript may be better off without the consumers' healthy diets section because technically the section fails to address their objective with respect to food security.

Thank you for this comment. We took it into account and removed the "meets end needs" part from the text. When it comes to the issue of diet, we wanted to take into account a specific indicator that shows consumer subjective perception vs. aggregated economic data, thus we left and rewritten the sub-chapter related to consumer ability to afford a meal with meat, chicken, fish or equivalent and prevalence of severe food insecurity. We hope this section shows a bit of a different, more subjective angle of our analysis. We also avoided calling a diet “healthy”. We stayed with the Eurostat definition in the entire paper.
